# The Mechanism of Zinc Sulfate in Improving Fertility in Obese Rats Analyzed by Sperm Proteomic Analysis

**DOI:** 10.1155/2020/9876363

**Published:** 2020-05-04

**Authors:** Jing Ma, Ruiyu Han, Yuanlong Li, Tong Cui, Shusong Wang

**Affiliations:** ^1^NHC Key Laboratory of Family Planning and Healthy, Hebei Key Laboratory of Reproductive Medicine, Hebei Research Institute for Family Planning Science and Technology, Shijiazhuang 050071, China; ^2^Graduate School of Hebei Medical University, Shijiazhuang 050017, China; ^3^School of Chemistry and Materials Science, Hebei Normal University, Shijiazhuang 050024, China

## Abstract

This study investigates the mechanism underlying the improving effect of zinc on fertility in obese rats using proteomics. The effects of three different doses of ZnSO_4_ on spermatogenesis and hormone levels were studied. Testicular spermatogenesis was observed by HE staining. Serum estrogen and testosterone levels were measured by chemiluminescent microparticle immunoassay. Sperm proteomic analysis was performed by liquid chromatography-mass spectrometry. The DAVID database was used to perform the GO enrichment analysis and KEGG pathway analysis of the differentially expressed genes, and the STRING online database was used to construct a PPI network. The sperm count, sperm motility, and testosterone hormones of the ZnSO_4_-treated rats group were increased. ZnSO_4_ improved testicular structure and spermatogenesis abnormalities caused by obesity. Proteomic analysis showed that there were 401 differentially expressed proteins in a total of 6 sperm samples from the ZnSO_4_-treated group and the obesity groups. Differential proteins were input into the DAVID website. The 341 identified proteins were then classified according to their biological functions. The KEGG analysis showed that the enriched signal pathways included glycolysis/gluconeogenesis, carbon metabolism, citrate cycle, fatty acid metabolism, and pyruvate metabolism. Some proteins were shown to be associated with valine, leucine, and isoleucine degradation pathways. STRING analysis obtained 36 node proteins. Cytoscape analysis showed that these proteins mainly participated in nine networks including metabolic process, oxidation-reduction, aerobic respiration, RNA splicing, and glutathione conjugation. ZnSO_4_ may improve the fertility of obese male rats by regulating protein expression related to metabolism, inflammation, and sperm maturation.

## 1. Introduction

Obesity is associated with male infertility. There is a certain time consistency among the increase of male infertility rate, the decrease of semen quality, and the increase of obesity rate [1]. Obesity leads to pathological changes in testicular ultrastructure, and the apoptosis of spermatogenic cells is significantly increased [2]. The decrease in the number of mature sperm may be one of the reasons leading to the low spermatogenic ability of obese people.

There are trace element metabolism disorders in obese people. The disturbed level of trace element metabolism in the body will induce corresponding effects on lipid metabolism. In the male reproductive system, zinc ions are mainly distributed in the testis, epididymis, prostate, and semen. Zinc is a marker of prostate function. Moreover, it regulates sperm function, acts as a cofactor for most enzymatic reactions, and helps maintain sperm motility. Zinc also plays an important role in testicular development and sperm formation [3]. Zinc deficiency significantly enhances apoptosis of germ cells in mouse testis and causes spermatogenesis arrest and fertilization damage [4]. Studies have shown that obese men are 3.5 times more likely to have oligozoospermia than men with normal weight [5, 6]. Zinc supplementation can reduce the weight of obese people. Blood glucose status (fasting blood glucose), blood lipid parameters (total cholesterol, triglyceride level, high-density lipoprotein cholesterol, and low-density lipoprotein cholesterol), and blood pressure are improved after zinc supplementation [7]. Oral zinc preparation can improve the content of zinc in seminal plasma, promote the transformation of sperm nuclear protein (i.e., from lysine to arginine), and inhibit the premature depolymerization of the sperm nucleus. It can improve sperm motility and semen quality of infertile patients without obvious side effects [8]. However, the application of proteomics in understanding the effects of ZnSO_4_ treatment on sperm proteins in obesity is still limited and further exploration is required.

In this study, the effects of three different doses of ZnSO_4_ on spermatogenesis and hormonal levels of obese rats were investigated. The mechanism underlying this effect was further analyzed by proteomic analysis.

## 2. Materials and Methods

### 2.1. Animals

The 7-week-old Sprague Dawley rats (weighing 180-200 g) were purchased from the Experimental Animal Center of Hebei Medical University. They were maintained on a 12 h dark/light cycle in an air-controlled room (temperature, 22.0 ± 10°C; humidity, 55 ± 5%) with free access to water and animal chow. All animal experiment procedures were approved by the Ethics Committee of the Hebei Institute of Family Planning Science and Technology.

### 2.2. Obesity Model Establishment, Animal Grouping, and Sampling

The rats were randomly divided into two groups: normal feed group (15 animals per group) and obesity model group (30 animals per group). Each group was fed the corresponding diets for 8 weeks, i.e., a normal chow diet for the normal group and a high-fat diet for the obesity model group. Rat body weights were weighed weekly and recorded for 8 weeks. The obesity model was considered successful when the average body weight of the model group was 1.2 times than that of the control group. The length of rats were measured (nose tip to the anus), and the Lee index was calculated by the formula Lee′s index = (weight × 1000)^^(1/3)^/body length (cm).

After establishment of the obesity model, the model rats were randomly divided into two groups: the obesity group and the ZnSO_4_-treated group. Rats in the ZnSO_4_-treated group received ZnSO_4_ (Tianjin Yongda Chemical Reagent Company Limited) (3.2 mg/kg/d) for 4 weeks by oral gavage. At the end of the experiment, the body weights, testicular weight, epididymal weight, and peritesticular fat of each group were measured, and blood was taken from the abdominal aorta. Sperm samples were harvested from the caudal epididymis. The testes were removed.

### 2.3. Sperm Count and Sperm Motility

The left epididymis of each rat was harvested immediately after sacrifice and was transferred to a tube containing 1 mL of warm (37°C) saline. They were then shaken at 37°C for 5 min to allow dispersal of spermatozoa. Approximately 10 *μ*L of diluted sperm suspension was transferred to each counting chamber of the hemocytometer to determine sperm concentration and motility. The motility was measured as the percentage of motile sperm (a+b grade) among total spermatozoa.

### 2.4. Determination of Fasting Serum Glucose, Blood Lipids, and Insulin

Total cholesterols, triglyceride, low-density lipoprotein, and high-density lipoprotein levels in serum were measured on a Siemens Centaur XP analyzer by a chemiluminescent microparticle immunoassay kit (Medical System Biotechnology Co., LTD). Fasting serum glucose was measured by a glucose detection kit (Medical System Biotechnology Co., Ltd., Ningbo, China) on an ACCUTE TBA-40FR analyzer (Toshiba Medical Systems Co., Tokyo, Japan). Serum levels of insulin were determined by chemiluminescence immunoassay on a UniCel DxI 800 system (Beckman Coulter, CA, USA) with corresponding reagents (Beckman Coulter, CA, USA).

### 2.5. Enzyme-Linked Immunosorbent Assay (ELISA)

Leptin level was determined by ELISA kits (Multisciences Biotech Co., Ltd., Hangzhou, China). After termination of the reaction, absorbance was read at 450 nm.

### 2.6. HE Staining

The testes were fixed in Bouin's solution overnight. The testes were then dehydrated using alcohol and embedded in paraffin. Samples were sectioned at 5 *μ*m thickness and stained with HE staining. Testicular spermatogenesis was observed under a light microscope.

### 2.7. Measurement of Androgen Hormones

Serum estrogen and testosterone levels were measured on a Siemens Centaur XP analyzer by chemiluminescent microparticle immunoassay. The detection kit was purchased from Siemens Healthcare Diagnostic Inc. and Cayman Chemical, Michigan, USA.

### 2.8. Liquid Chromatography-Mass Spectrometry

The sperm protein samples used in this study were from the three groups (normal group, obesity model group, and ZnSO_4_-treated group). Sperm samples were harvested from the caudal epididymis. Briefly, proteins were extracted with lysate buffer with 8 M urea, 10 mM DTT, and protease inhibitor. Sonication was performed for 3-5 min. The supernatant was collected after 20000 g centrifugation for 10 min at 4°C, and protein was quantified with the Bradford method. The extracted proteins were incubated with 100 mM TEAB to 100 *μ*L and then with 200 mM TCEP at 55°C for 1 h. After that, 5 *μ*L of 375 mM iodoacetamide (IAA) was added. After incubation in the dark for 30 min, precooled acetone was added and it was incubated overnight at -20°C. The supernatant was removed carefully after 8000 g centrifugation at 10°C for 10 min, and the lysate was left at room temperature for 2-3 min to dry. Finally, 100 *μ*g of protein, 100 *μ*L of 100 mM TEAB solution, and trypsin enzyme ratio protein (1 : 50) were mixed together and the enzyme digestion was performed overnight at 37°C.

Liquid chromatography-mass spectrometry: partially digested samples were taken and dissolved in solution A (2% ACN/98% H_2_O/0.1% FA). After centrifugation at 20000 g for 30 minutes, the supernatant was taken and the protein sequence was detected by EASY-nLC liquid phase-Q Exactive mass spectrometer (American Thermo Fisher).

Mass spectrometry conditions were 90 min for data acquisition time, 2 kV for spray voltage, 320°C for capillary temperature, 27% for normalized collision energy, and 300-1400 Da for collection mass range. Primary parameters were 70000 for resolution, 3*e*6 for AGC target, 60 ms for maximum IT, and profile for spectrum data type. Secondary parameters were 17500 for resolution, 5*e*4 for AGC target, 80 ms for maximum IT, and 3.0 m/z for isolation window.

### 2.9. Data Retrieval

In the MaxQuant 1.5.2.8 search engine, the first error is 20 ppm, the second error is 0.02 Da. The fixed modification is as follows. Cysteine is modified to Carbamidomethyl-Cys, and the variable modification is as follows: Oxidation-M, LysisC or Trypsin, or Glu-C digestion. Enzymatic digestion allows up to 2 missing sites. Data gap filling, normalization, and difference screening (*P* < 0.05%) were all performed using the Perseus software standard settings. A total of 1344 proteins were identified and quantified in 6 samples from both groups. Qualitative and quantitative information of Zn and G group on differential proteins were obtained. Perseus software performed *t*-test and significance analysis on the quantitative results and ratios of proteins. The obtained differential protein list is as follows: a total of 401 significant differential proteins were obtained by *t*-test results and differential distribution analysis results.

### 2.10. GO (Gene Ontology) and KEGG (Kyoto Encyclopedia of Genes and Genomes) Analysis

Differential protein was imported into the DAVID (Functional Annotation Bioinformatics Microarray Analysis) website (https://david.ncifcrf.gov/) for basic bioinformatics extraction. The web tools provided by the DAVID were used to search for functional annotation terms and pathways that were enriched in the above-identified proteins, including cellular component, molecular function, and biological process.

### 2.11. Protein Interaction Network Analysis

The differential proteins screened were imported into STRING (https://string-db.org/) online database for analysis. The differential gene interaction network map was drawn. The interactive network data was exported to the Cytoscape 3.2 software to determine the network center node protein.

### 2.12. Statistical Analysis

Data were displayed as mean ± standard error of the mean. The statistical analysis was performed in SPSS22.0 using one-way analysis of variance (ANOVA) with a *P* value < 0.05 considered statistically significant.

## 3. Results

### 3.1. Semen Parameters and Testosterone Hormone Level Changes in Sperm after ZnSO_4_ Treatment

Compared with the control group, the body weight, peritesticular fat, Lee's index, total cholesterols, triglyceride, high-density lipoprotein, and leptin of obesity group rats and leptin of ZnSO_4_-treated group rats increased significantly. Compared with the control group, the low-density lipoprotein of obesity group rats decreased significantly. Compared with the obesity group, the body weight, peritesticular fat, and Lee's index decreased in the ZnSO_4_-treated group, and the difference was statistically significant (Table 1). In order to detect the ZnSO_4_ effects on the fertility of rats, each group of semen parameters was first evaluated according to the WHO 2010 criteria [9]. The number of sperm and sperm motility were inhibited in the obesity group as shown in Table 2. Compared with the obesity group, the sperm count and sperm motility of the ZnSO_4_-treated rats increased, suggesting that ZnSO_4_ improves semen parameters in obese rats. Obesity itself can cause an increase in blood lipids, but our results showed that blood glucose, blood lipids, and insulin levels did not reach the level of diabetes. It can be considered that the confounding factors of diabetic complications were excluded. Furthermore, we detected serum testosterone level. The results showed that testosterone hormones increased in the ZnSO_4_-treated group compared with the obesity group (Table 2). Thus, ZnSO_4_ treatment could improve semen quality of obese rats.

### 3.2. ZnSO_4_ Treatment Improves the Recovery of Testicular Impairment Induced by Obesity

**Figure 1:**
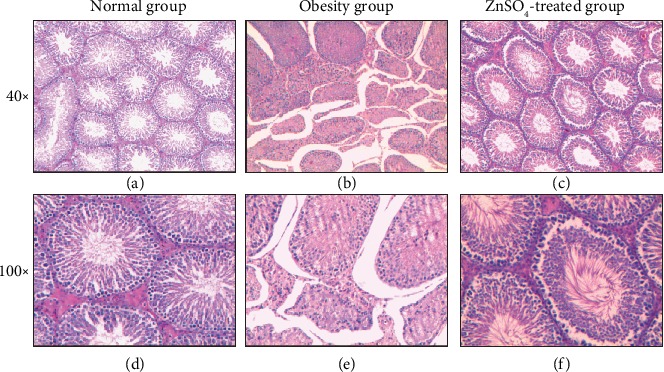
Cross-sectional morphology of the testes for each group at magnification (a–c) 40x and (d–f) 100x by HE staining: (a, d) normal group, (b, e) obesity group, and (c, f) ZnSO_4_-treated group.

Subsequently, we conducted histology analysis of testicular tissue and the results were shown in Figure 1. According to testis histology, the normal group showed normal spermatogenesis (Figures 1(a) and 1(d)), whereas the obesity group showed disrupted spermatogenesis as the lumen of seminiferous tubule was almost empty (Figures 1(b) and 1(e)). As we expected, the ZnSO_4_-treated group showed significant improvement compared with the obesity group in the testis histology with the appearance of normal Sertoli and Leydig cells and undisrupted spermatogenesis (Figures 1(c) and 1(f)). Thus, ZnSO_4_ can improve testicular structure and spermatogenesis abnormalities caused by obesity.

### 3.3. Classification of 341 Sperm Proteins by Bioinformatics: Cellular Component, Molecular Function, and Biological Process

**Figure 2:**
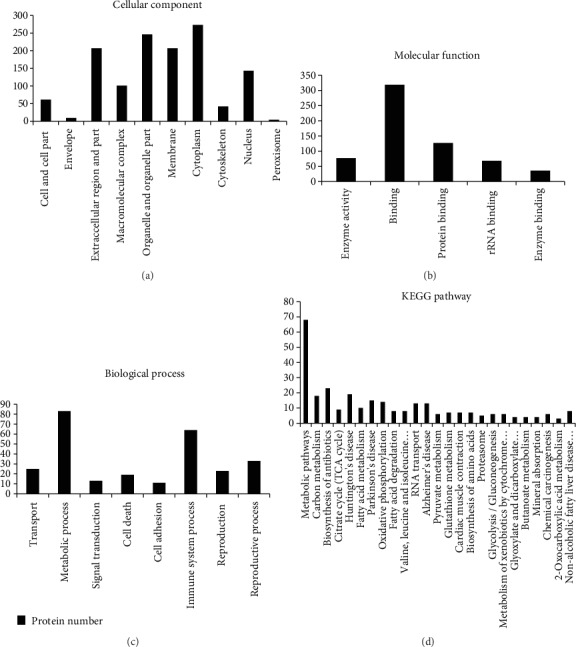
Functional classification and enrichment of identified proteins in sperm. GO function classification analysis of total identified proteins in sperm according to their (a) cellular component, (b) molecular function, and (c) biological process. (d) KEGG pathway enrichment analysis of total identified proteins in sperm.

To determine the differentially expressed proteins, proteomic analysis was performed. A total of 1344 proteins were identified and quantified in a total of 6 sperm samples from the ZnSO_4_-treated group and the obesity group. Perseus software performed *t*-test and differential significance analysis on the quantitative results and ratios of proteins. A total of 401 significant proteins were obtained. Differential proteins were input into the DAVID website for the ZnSO_4_-treated group and the obesity group differences in protein function. In GO classification, 371 proteins were analyzed, and 30 proteins did not correspond. The 341 identified proteins were then classified according to their biological functions. We used the web tools provided by the DAVID to search for functional annotation terms and pathways that were enriched in the above-identified proteins. The results of these analyses were shown in Figure 2. We focused on the ontology of cellular component, molecular function, and biological process for functional annotation term enrichment analysis with *P* < 0.005 and ratio > 2.

In the “cellular component” group (Figure 2(a)), the category analysis showed that 59% of the proteins with significant differences were organelle components, and 60.7% of those were organelle constituents. In addition, 29.6% of the proteins belonged to a macromolecular complex. The “molecular function” GO term analysis revealed that 22% of the proteins were classified as proteins with catalytic activity (Figure 2(b)). The other proteins could be classified as protein binding, rRNA binding, and enzyme binding. In terms of the “biological process” database (Figure 2(c)), the majority of the 24% proteins were associated with metabolic process. Besides, proteins were linked with transport, signal transduction, cell death, cell adhesion, immune system process, and reproduction. The signal pathway analysis results (Figure 2(d)) with concentrated protein and enrichment are as follows. Multiple metabolic pathways such as glycolysis/gluconeogenesis, carbon metabolism, citrate cycle (TCA cycle), fatty acid metabolism, and pyruvate metabolism have been disturbed and affected, and some proteins have been shown to be associated with valine, leucine, and isoleucine degradation pathways.

### 3.4. Zinc Effects Are Further Identified by Differentially Expressed Sperm Proteins

**Figure 3:**
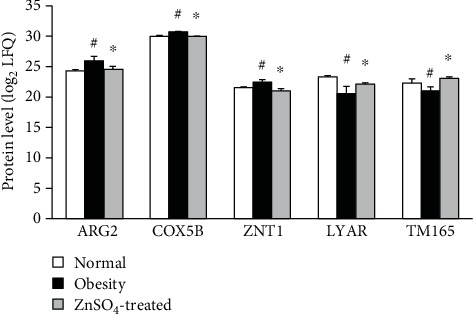
Quantification analysis showed six significantly expressed proteins. These proteins include ARG2, COX5B, ZNT1, LYAR, and TM165. ^#^*P* < 0.05 compared to normal control; ^∗^*P* < 0.05 compared to obesity.

Quantification analysis was performed to compare protein levels between the three groups. In the differential proteins, we selected metabolic, zinc transport-associated proteins and node proteins in the network (Table 3). Proteins with statistically significant changes were shown in Figure 3. These proteins were ARG2, COX5B, ZNT1, LYAR, and TM165. Compared with the obesity group, the expression of ARG2, COX5B, and ZNT1 in the ZnSO_4_-treated group was significantly decreased, while the expression of LYAR and TM165 was significantly increased.

### 3.5. The Differential Protein Interaction Network Is Established Using the STRING Network Database

**Figure 4:**
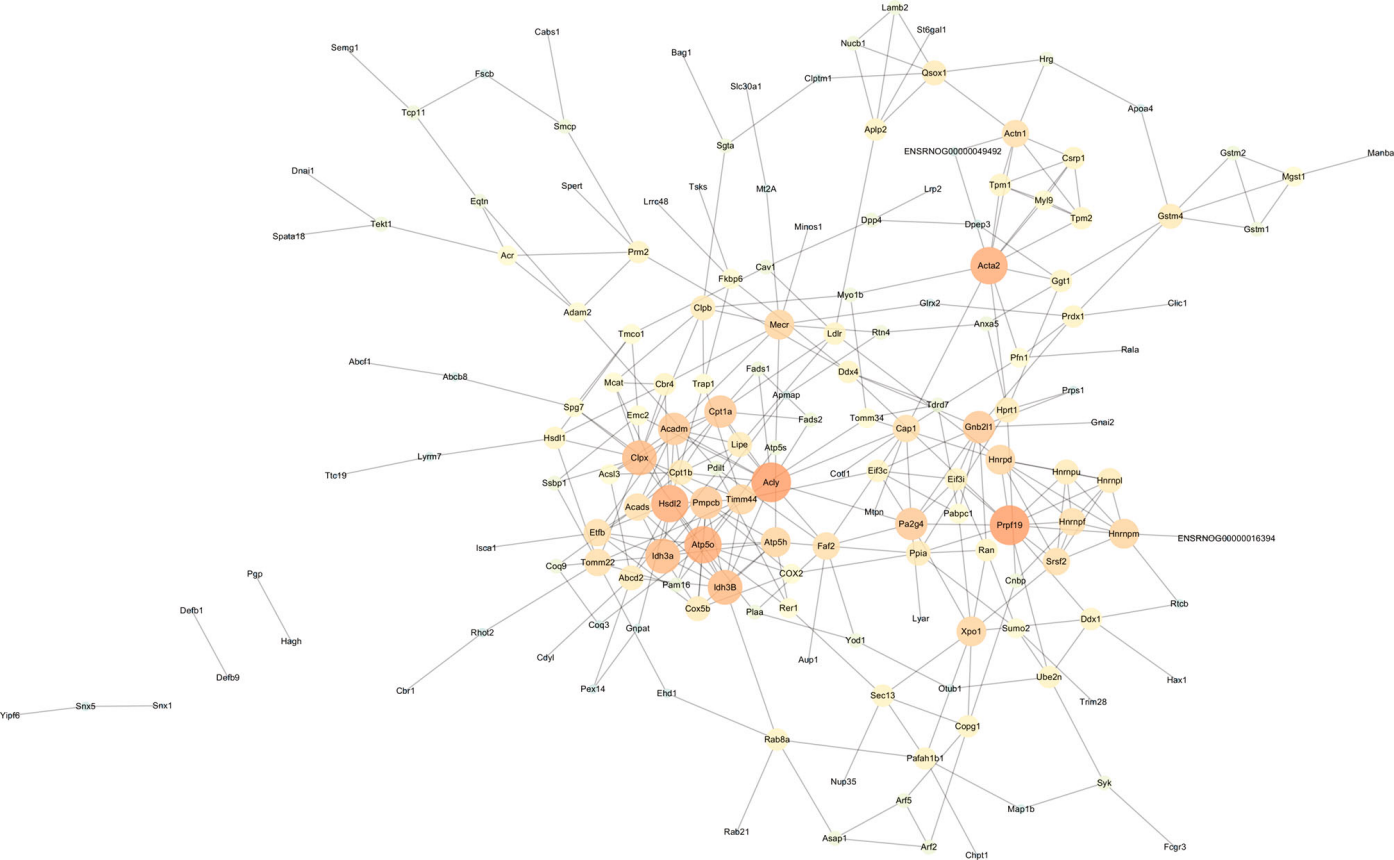
STRING protein interaction network. The circle represents the gene and the line represents the relationship between the genes.

STRING is an online analysis software that analyzes and predicts the interaction between known proteins. STRING software establishes a scoring mechanism to make corresponding weights on different sources of data and finally gives a comprehensive score and then constructs a network map of protein-protein interactions [10]. The 341 differential proteins screened were imported into STRING (http://string-db.org/) online database for analysis, and 341 proteins were identified, and the differential gene interaction network map was generated. After that, the interactive network data was exported to the Cytoscape 3.2 software to determine the network center node protein. It can be seen that the network of differential protein composition is complex (Figure 4). We then used the Cytoscape plugin to analyze the node proteins in the network, and a total of 36 node proteins were obtained from the analysis (Table 3). From a list of top networks generated using STRING, we selected the subnetworks. Cytoscape analysis showed that these proteins mainly participated in nine networks including metabolic process, oxidation-reduction, aerobic respiration, RNA splicing, and glutathione conjugation.

## 4. Discussion

The WHO defines a person with abnormal or excessive fat accumulation as overweight or obese, and this state constitutes a growing threat to the health of people globally [11, 12]. Some reports show that the rate of obesity is increasing rapidly [13, 14], which not only increases the risk of diseases but also in parallel increases patients' risk of developing reproductive disorders. As the reproductive function of men deteriorates globally [15–17], more and more people have realized that obesity decreased semen quality. With increased BMI, the semen parameters are changed, thus changing the physical and molecular structure of spermatozoa [18, 19]. Previous studies have found that sperm concentration and total motile sperm count were detrimentally affected by a high BMI [20, 21]. In the present study, rats in the obesity groups showed a significant decrease in sperm concentration and sperm motility of sperm compared with those in the normal weight group, whereas ZnSO_4_ did improve semen parameters compared to the obesity group.

Imbalances in sex hormones may affect male reproduction, and an overweight status may affect hormone levels in men [20, 21]. Simultaneously, studies have shown that obesity is closely related to endocrine disorders, such as sex hormone abnormalities [22, 23]. Obesity in men has a negative impact on male reproductive potential because of changes in hormone levels [24]. Therefore, we tested the serum levels in each group. Testosterone hormones increased in the ZnSO_4_-treated group compared with the normal and obesity groups. It appears that the ZnSO_4_ treatment significantly increased the androgen hormone levels to match the normal control group level. Reduced body weight and blood lipid level in ZnSO_4_-treated rats may repair the Leydig cells, thus increasing the testosterone level. As a consequence, a functional male reproductive system can be regenerated, assisting spermatogenesis and the testicular structure regeneration [25]. Evidently, the testis histology of the ZnSO_4_-treated group has been improved with Sertoli and Leydig cells regenerated and the sperm in the lumen restored. Therefore, a large-scale comparative proteomics provides an effective approach to identify any protein expression difference between the obesity and ZnSO_4_-treated groups. Our study identified the differences in protein expression profiles between normal fertile sperm and sperm from ZnSO_4_-treated groups.

GO annotation analysis showed that the 24% proteins were associated with metabolic process and 22% of the proteins were classified as proteins with catalytic activity. The other proteins were classified as protein binding, rRNA binding, and enzyme binding, including ATP binding. It is well known that ATP-binding proteins play a fundamental role in biological processes, which indicates changes in synthetic and metabolic processes. Mitochondria are organelles that provide energy (ATP) to cells. Mitochondria are also the primary target of oxidative stress. In the male body, mitochondria are the main energy plant in the process of spermatogenic cell maturation and also provide energy for the spermatozoa after ejaculation. Therefore, when oxidative stress occurs in obese men, mitochondria in sperm can be greatly damaged. Sperm is susceptible to oxidative stress and lacks the ability to repair damage. High-fat diet induces oxidative stress in obese rats, which induces damage to sperm mitochondrial membrane and affects mitochondrial function [26]. Egwurugwu et al. [27] concluded that zinc sulfate had some significant positive effects on androgen and sperm quality at physiological doses. However, it was harmful at higher doses.

ARG2 is known to localize in mitochondria [28]. It also plays a crucial role in the production of ornithine, which is a precursor of proline, hydroxyproline, and polyamine, and is essential for cell proliferation. Obesity and its associated diseases are characterized by low levels of chronic inflammation [29, 30]. ARG2 promotes proinflammatory responses in macrophages and contributes to evidence of atherosclerosis and obesity-related insulin resistance [31]. We believe that early obesity may lead to upregulation of arginase, resulting in systemic changes in arginase and arginine metabolites. Upregulation of ARG2 in the obese group may be associated with cell proliferation and chronic inflammation caused by obesity. Arginase improves obesity-induced liver lipid and systemic fat abnormalities by inhibiting activation of pathways involved in hepatic triglyceride metabolism and mitochondrial function [32, 33].

Of these proteins, COX5B particularly is of high interest and linked to mitochondrial function and cellular energy production [34]. Cytochrome oxidase (COX, Complex IV) is a mitochondrial electron transport chain enzyme that resides in the mitochondrial inner membrane, and its activity is required to generate the proton motive force that drives downstream ATP synthesis [35]. It is one of three mitochondrial isoforms of cytochrome oxidase, that is, the Complex IV of the mitochondrial respiratory chain. COX5B is involved in the final step of the oxidative phosphorylation, with the production of H_2_O, and the maintenance of the electrochemical gradient needed to produce ATP. Thus, reduced levels of ARG2 and COX5B in zinc sulfate-treated rats may suggest a zinc-induced effect on fertility in obese rats, especially in testicular regeneration, spermatogenesis, and sperm motility.

Some differentially expressed proteins identified in this study are involved in the zinc transport process. For example, Elgazar et al. [36] found that ZnT1 is present in the plasma membrane and the cytoplasm of the supporting cells. Studies have shown that Znt1 plays an important role in zinc homeostasis in adult mice [37]. Metal-responsive transcription factor-1 (MTF-1) plays a role in coordinating cellular responses to metal homeostasis and oxidative stress. MTF-1 is a zinc-dependent transcription factor that stimulates metallothionein and zinc transporter-1 (ZNT-1) genes with increasing zinc concentration [38]. Foster et al. [10] showed that the relative expression of zinc transporter mRNA was very variable. ZnT1 is the most abundant in the testis, and it has interactions in zinc transport across the plasma membrane. Noh et al. [39] reported that ZnT1 mRNA levels were slightly elevated in obese women, and zinc transporter changes may also be associated with obesity-related inflammatory states.

The Ly1 antibody reactive homolog (LYAR) was first described by Su et al. as a cDNA encoding zinc finger protein isolated from mouse T-cell leukemia line [40]. The *Lyar* gene, which is known to be expressed abundantly in the testis, encodes a nucleolar protein that contains a LYAR-type C2HC zinc finger motif and three nuclear localization signals. Lee et al. [41] found that the LYAR protein was present in spermatocytes and spermatids, but not in sperm. However, we detected LYAR expression in sperm, and its expression decreased in sperm of obese rats and increased in ZnSO_4_-treated groups. LYAR is identified to be associated with cytoplasmic ribosomes in male germ and cancer cells and is involved in preribosomal RNA processing within the nucleus [42]. LYAR is a modulator of one of the two basic steps of translation initiation in mammalian male germ cells and certain types of tumors [43]. LYAR considerably suppresses the transcription of oxidative stress genes, including SLC7A11, HMOX1, and CHAC1. Myc oncoprotein upregulates LYAR expression by activating its gene transcription, and the upregulation of LYAR, in turn, protects cancer cells against oxidative stress-mediated apoptosis through reducing CHAC1 gene expression [44].

Transmembrane protein 165 (TM165) is a Golgi transmembrane protein [45], and its deficiency causes a congenital disorder of glycosylation. TM165 is both transcriptionally and translationally overexpressed in hepatocellular carcinoma and associated with invasive ability of hepatocellular carcinoma [46]. However, data obtained in recent study give several indications of their implication in calcium and manganese homeostasis [47]. TM165 supplies Ca2+ and Mn2+ to the Golgi complex in exchange for H+ to sustain the functions of lactose synthase and potentially other glycosyltransferases [48, 49]. The human Golgi protein TM165 can transport calcium and manganese in yeast and bacterial cells [50]. Our study found that TM165 expression in obese rats decreased and increased after zinc supplementation, suggesting that TM165 increased after zinc supplementation.

## 5. Conclusions

In conclusion, the results of this study provide evidence that ZnSO_4_ may improve hormone levels, testicular regeneration, and fertility. Proteomic analysis further shows that ZnSO_4_ may improve the fertility of obese male rats by regulating protein expression related to metabolism, inflammation, sperm maturation, and other interactions.

## Figures and Tables

**Table 1:** Comparison of normal, obesity, and ZnSO_4_-treated groups in baseline data.

	Normal	Obesity	ZnSO_4_-treated
Body weight (g)	298.09 ± 29.31	340.90 ± 44.74^#^	280.41 ± 16.85^∗^
Testicular weight (g)	1.80 ± 0.81	2.21 ± 0.52	1.60 ± 0.73
Epididymal weight (g)	0.88 ± 0.15	0.98 ± 0.12	0.85 ± 0.13
Peritesticular fat (g)	2.60 ± 0.61	3.66 ± 0.92^#^	2.59 ± 0.62^∗^
Body length (cm)	23.44 ± 1.07	23.32 ± 1.63	23.00 ± 0.69
Lee's index	0.28 ± 0.01	0.30 ± 0.02^#^	0.28 ± 0.01^∗^
Total cholesterols (mmol/L)	1.38 ± 0.18	1.80 ± 0.26^#^	1.48 ± 0.16^∗^
Triglyceride (mmol/L)	0.48 ± 0.07	0.58 ± 0.05^#^	0.51 ± 0.03^∗^
High-density lipoprotein (mmol/L)	0.40 ± 0.09	0.53 ± 0.05^#^	0.44 ± 0.05^∗^
Low-density lipoprotein (mmol/L)	0.57 ± 0.13	0.45 ± 0.04^#^	0.57 ± 0.04^∗^
Fasting serum glucose (mmol/L)	8.72 ± 2.43	8.69 ± 1.36	8.29 ± 2.25
Insulin (mU/L)	19.75 ± 2.83	22.00 ± 3.23	20.85 ± 2.97
Leptin (pg/mL)	177.83 ± 31.51	258.23 ± 46.95^#^	231.26 ± 49.11^#^

Note: ^#^*P* < 0.05 compared to normal control; ^∗^*P* < 0.05 compared to obesity.

**Table 2:** Semen parameters and testosterone hormone levels of normal, obesity, and ZnSO_4_-treated groups.

	Normal	Obesity	ZnSO_4_-treated
Sperm concentration (9 × 10^6^ per mL)	28.38 ± 8.63	17.50 ± 4.23^#^	26.29 ± 8.73^∗^
Sperm motility (a+b%)	16.75 ± 6.21	8.50 ± 4.51^#^	15.86 ± 7.06^∗^
Testosterone (ng/mL)	1.73 ± 1.50	2.51 ± 2.03	6.01 ± 4.34^#∗^
Estrogen (pg/mL)	24.22 ± 2.89	23.07 ± 1.96	26.15 ± 2.90

Note: ^#^*P* < 0.05 compared to normal control; ^∗^*P* < 0.05 compared to obesity.

**Table 3:** STRING protein interaction network nodes.

Cluster	Score (density∗#nodes)	Nodes	Edges	Node
1	5.2	11	26	IDH3B, PMPCB, IDH3A, ATP5O, ATP5H, COX5B, ACADM, ACLY, LIPE, CPT1B, CPT1A IDS
2	5	5	10	HNRNPF, PRPF19, HNRNPU, HNRNPM, SRSF2
3	4	4	5	GSTM2, GSTM4, GSTM1, MGST
4	4	4	6	QSOX1, APLP2, NUCB1, LAMBB2
5	3.333	4	5	ADAM2, EQTN, ACR, PRM2
6	3	3	3	ANXA5, HPRT1, GGT1
7	3	3	3	ARF5, ASAP1, ARF2
8	3	3	3	SEC13, PAFAH1B1, XP01

## Data Availability

The data used to support the findings of this study is available upon request.
